# Bioturbation enhances the aerobic respiration of lake sediments in warming lakes

**DOI:** 10.1098/rsbl.2016.0448

**Published:** 2016-08

**Authors:** Viktor Baranov, Jörg Lewandowski, Stefan Krause

**Affiliations:** 1 Leibniz-Institute of Freshwater Ecology and Inland Fisheries, Müggelseedamm 310, 12587 Berlin, Germany; 2 School of Geography, Earth and Environmental Sciences, University of Birmingham, Edgbaston, Birmingham B15 2TT, UK; 3 Faculty of Mathematics and Natural Sciences, Geography Department, Humboldt University of Berlin, Rudower Chaussee 16, 12489 Berlin, Germany

**Keywords:** temperature, bioturbation, lakes, Chironomidae, respiration, carbon metabolism

## Abstract

While lakes occupy less than 2% of the total surface of the Earth, they play a substantial role in global biogeochemical cycles. For instance, shallow lakes are important sites of carbon metabolism. Aerobic respiration is one of the important drivers of the carbon metabolism in lakes. In this context, bioturbation impacts of benthic animals (biological reworking of sediment matrix and ventilation of the sediment) on sediment aerobic respiration have previously been underestimated. Biological activity is likely to change over the course of a year due to seasonal changes of water temperatures. This study uses microcosm experiments to investigate how the impact of bioturbation (by Diptera, Chironomidae larvae) on lake sediment respiration changes when temperatures increase. While at 5°C, respiration in sediments with and without chironomids did not differ, at 30°C sediment respiration in microcosms with 2000 chironomids per m^2^ was 4.9 times higher than in uninhabited sediments. Our results indicate that lake water temperature increases could significantly enhance lake sediment respiration, which allows us to better understand seasonal changes in lake respiration and carbon metabolism as well as the potential impacts of global warming.

## 1. Background

**Figure 1.**
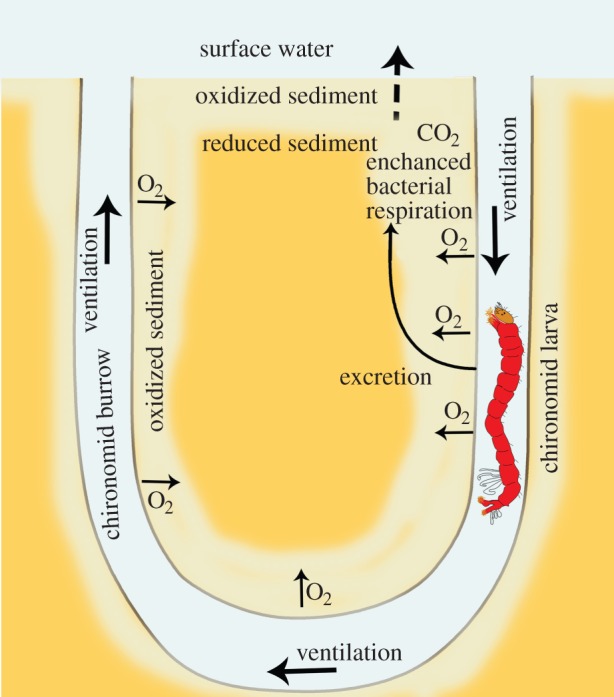
The impacts of chironomid larvae's (Diptera, Chironomidae) bioturbation on sediment biogeochemistry. (Online version in colour.)

Bioturbation is one of the least studied drivers of sediment respiration in lakes [1]. Bioturbation is defined as “all transport processes carried out by animals that directly or indirectly affect sediment matrices. These processes include both particle reworking and burrow ventilation” [2]. Bioturbation by freshwater animals, especially chironomid larvae (Diptera, Chironomidae), mayfly larvae (Ephemeroptera, Ephemeridae) and tubificid worms (Oligochaeta, Tubificidae) is capable of increasing the respiration of freshwater sediment by up to five times [3,4]. Only a small portion of this increase has been found to result from the respiration of the bioturbating animals themselves (approx. 10–20%), with the remainder being attributed to the enhancement of sediment bacterial aerobic metabolism [3,5]. Chironomid larvae are also known as bloodworms (Diptera, Chironomidae) [6] as they possess haemoglobin and are red-coloured. Representatives of the family Chironomidae are among the most important freshwater bioturbators. They have complex and long-lasting impacts on nutrient cycling at the sediment–water interface due to sediment-redistribution, modification of sediment microstructure, burrow ventilation, sediment oxidation (bioirrigation) and enhanced bacterial activity in the sediment (figure 1) [7,8]. Owing to their large densities especially in eutrophic water bodies, their burrowing and ventilation activities can dramatically impact freshwater biogeochemistry [6]. For example, a volume equivalent to the total water volume of the shallow Lake Müggelsee (Germany, mean depth 5 m) is pumped by chironomids through their burrows once a week [6].

High water temperatures in temperate regions frequently cause algal blooms, resulting in organic matter inputs into benthic ecosystems which can be beneficial for filter feeders and detritus-collecting benthic organisms such as *Chironomus plumosus* L., 1758 that are able to tolerate low oxygen concentrations due to their capability of oxyregulation and respiration in low oxygen environments [9]. Furthermore, warm summer water results in faster development, shorter life cycles, additional generations per year and higher reproduction rates—all resulting in higher animal densities and intensified turnover [10,11]. Although bioturbation of chironomids is temperature-dependent [1], previous studies largely ignored the influence of water temperature on their bioturbation [2,12]. Only few studies [9,10] have acknowledged that respiration of chironomid larvae themselves can be correlated with temperature [11]. It is commonly accepted that the locomotory activity of chironomid larvae increases with rising temperature. Pumping rates of larvae, which are important for ventilation of the burrows and larval respiration, also increase with rising temperatures [1,13]. Therefore, we hypothesize that temperature-enhanced chironomid densities in the benthic zone and their increased bioturbation activity may result in increased sediment aerobic respiration.

To test this hypothesis, we conducted lake sediment microcosm experiments with different larval densities (0, 1000, 2000 larvae m^−2^) and exposed them to a range of temperatures. The experiments deployed the resazurin–resorufin smart bioreactive tracer system to investigate the differences of respiration between set-ups with different densities and temperatures [14,15]. Decay of the bioreactive tracer resazurin is proportional to environmental respiration (especially sediment respiration under oxic conditions) (*r*^2^ = 0.88–0.99) [15]. Thus, resazurin can be used for the relative assessment of temperature-dependent differences in sediment respiration. Moreover, as we have shown in previous research [5], resazurin is not susceptible to respiration of apneustic aquatic animals (those receiving oxygen by means of diffusion through the water-impenetrable cuticle, such as chironomid larvae). Hence, the application of the smart tracer system allows the sediment respiration impacts of chironomid bioturbation to be quantified independently from their own respiration. Thus, we can separate the impacts of these two processes, which until recently presented an enduring problem in attempts to quantify bioturbation impacts on sediment respiration [4].

## 2. Material and methods

Experiments were conducted in cylindrical glass microcosms with a total volume of 566 ml (20 cm high, diameter 6 cm), containing 200 g of sediment from Lake Müggelsee (Berlin, Germany; sediment: black, muddy, organic-rich, water content 90 ± 3 (arithmetic mean ± s.d., *n* = 3), loss of ignition 76.7 ± 2.26 (arithmetic mean ± s.d., *n* = 3), total phosphorus 2.7 ± 0.5 mg (g DW)^−1^ (DW, dry weight), total nitrogen 10.1–16.8 mg (based on Kozerski & Kleeberg [16] and own measurements of the sediment DW, LOI)) and 250 ml bank filtrate from the same lake, which was obtained from waterworks in the vicinity of the lake. Overlying water in the microcosm was constantly aerated to assure homogeneous mixing and continuously oxic conditions in the water column overlying the sediments. All chironomid larvae used in the experiments belonged to the widely abundant species *Chironomus plumosus* L., 1758. All animals were of similar age (4th instar) and comparable size (20–22 mm). They were used in three different densities, with 0, 3 and 6 larvae per microcosm corresponding with zero, medium and high larvae density of 1000 and 2000 m^−2^. These densities correspond to *in situ* analysis of Lake Müggelsee sediment in 2014–2015 (*n* = 8), which revealed densities of 500 to 2000 specimens per m^2^; numbers are also in line with previous observation in the same lake [1]. Microcosm experiments in this study were conducted in a climate chamber (Binder kbf 720) at 5, 10, 15, 20 and 30°C.

Because of the relatively short duration of the experiment (duration of 8 days from the animal placement in the microcosms to the end of the experiment), no mortality was recorded (animals were counted before and after the experiment). We monitored temperature in the microcosms before and during the measurements. Animals were acclimatized to each respective temperature for 5 days prior to the start of the experiment. Also we considered that we had to have stable redox conditions in the sediment before starting the experiment. We have observed that oxic interfaces visible as light reddish brown coloration in sediments usually form between 24 and 36 h, well below the 5 days long pre-experimental phase, which was hence assumed to be sufficient.

At each temperature four replicates (microcosms) were conducted for each larval density.

Resazurin is a bioreactive tracer; its decay is proportional to aerobic respiration in the system (average *r*^2^ = 0.986) [14,15]. Resazurin and its daughter compound resorufin are fluorescent. We used a GGUN-FL30 fluorometer (Albilla Sarl, Switzerland) to quantify the fluorescent compounds in this experiment. The raz/rru smart tracer system provides a reliable proxy for oxygen consumption in the system as it directly measures the amount of aerobic respiration itself instead of analysing oxygen uptake in the water column [14]. Hence the raz/rru smart tracer system is well suited for respiration measurements in non-sealed microcosms, avoiding artefacts and shortcomings that are frequently observed in methods involving the analysis of oxygen consumption in sealed systems. The fluorescence-based detection of the raz/rru smart tracer system is highly sensitive to turbidity and concentration changes of particulate organic carbon. Thus, we filtered the samples before analysis (30 mm cellulose acetate syringe filter, pore diameter = 0.45 µm) [14]. Samples for measuring resazurin turnover rates were taken from each microcosm 5 and 8 h after tracer injection (with measurements taken immediately after injection used as baseline). We assessed the difference in raz/rru turnover between treatments, using ANCOVA; ln(rru/raz + 1) was used as response and temperature was used as covariate.

## 3. Results

**Figure 2.**
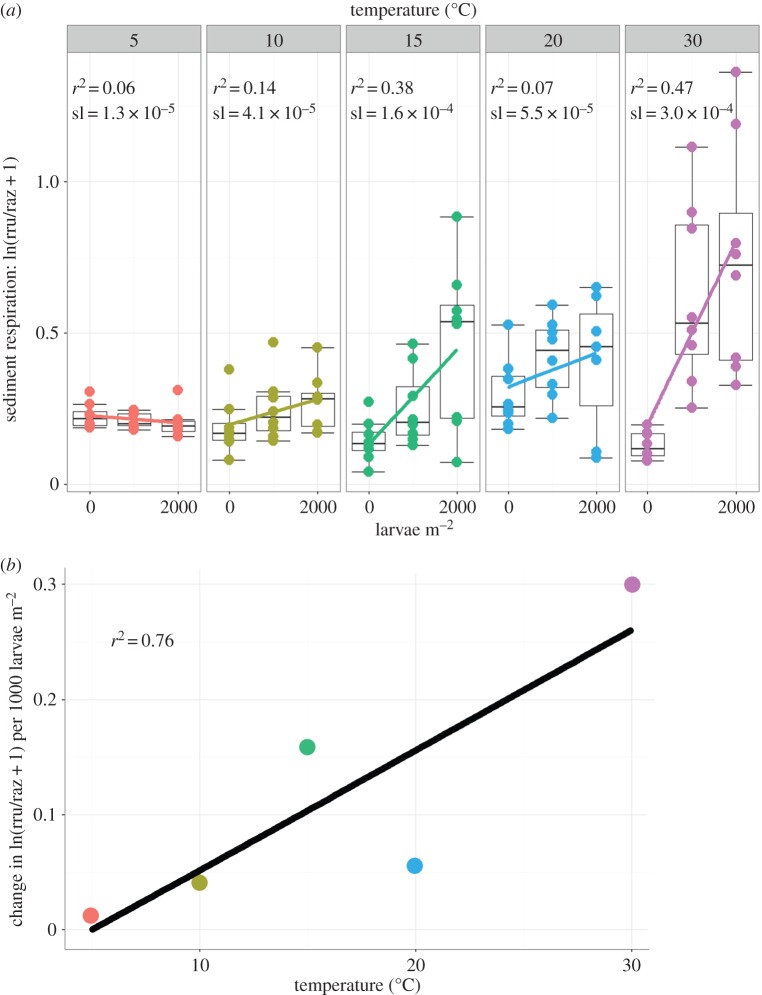
(*a*) The impact of increasing chironomid larval densities on sediment respiration at different temperatures, with raz being the concentration of resazurin and rru the concentration of resorufin, and ln(rru/raz + 1) indicating resazurin turnover (*n* = 8 for each density and temperature). Differences between sediment respiration rates for different larval densities and at different temperatures are indicated by the slopes (sl) of linear regression lines. (*b*) The water temperature regulates the impact of chironomid larvae's bioturbation on sediment respiration. The slopes of the regression lines from (*a*), showing the impact of larval density on respiration at a given temperature, are plotted against experimental temperatures. (Online version in colour.)

The microcosm experiments revealed that respiration differences between bioturbated and non-bioturbated sediments increased with rising temperature (figure 2*a*). At 5°C, the difference in sediment respiration between bioturbated and non-bioturbated microcosms was statistically not significant (ANCOVA, *p* > 0.05). At 10°C and above, respiration differences between non-bioturbated and chironomid-bioturbated sediments were statistically significant, and in fact increased with rising temperatures. Maximum differences between non-bioturbated and bioturbated sediment respiration were observed at 30°C, with respiration in microcosms with 1000 larvae m^−2^ being 4.4 times higher than in non-bioturbated sediments. Respiration in microcosms with 2000 larvae m^−2^ exceeded that of non-bioturbated sediments by six times. While temperature-dependent respiration increases were highly significant in microcosms with 1000 and 2000 larvae m^−2^ (*p* < 0.05) (figure 2*a*), there was no statistically significant relationship found for non-bioturbated microcosms (*p* > 0.05).

## 4. Discussion

The results of our experiments confirm the findings of previous studies, which reported increases of 20–300% in sediment respiration due to bioturbation. While direct respiration of chironomids is considered to be lower than the respiration of the bioturbated sediments [5,6,9–12], some authors have attributed up to 20% of the total respiration to chironomid respiration itself [4]. As mentioned above, the novel smart tracer system applied for measuring system respiration is not affected by chironomids' respiration; hence, for the first time we believe, increased respiration rates shown in the present study can be attributed solely to bioirrigation-impacted sediment respiration [5].

In order to analyse how the impact of larval density on sediment respiration scales with water and sediment temperatures, the change in resazurin turnover ln(rru/raz + 1) per larva (as indicated by the slopes of linear regressions in figure 2*a*) was compared against the investigated temperature range (figure 2*b*). As indicated by a strong positive correlation (*r*^2^ = 0.76), increasing temperatures significantly enhance the impact of chironomid bioturbation on sediment respiration, i.e. there are strong seasonal changes of sediment respiration in bioturbated sediments due to seasonal changes of lake temperatures, often covering ranges of more than 20°C. While the projected rise of temperatures of surface waters due to climate change is much lower than the range tested by us, as our data show, even a modest rise of lake water temperatures of a few degrees might impact bioturbation. Further investigations are required in order to clarify this matter.

This study reveals that high densities of chironomids in shallow lakes can significantly intensify sediment respiration, in particular, in warm and well-oxygenated systems. This effect is most pronounced in shallow, non-stratified lakes. In deeper lakes, increasing water temperatures will rather extend the duration of thermal stratification, causing the water above the sediment to become anoxic for longer periods and thus reduce chironomid activities such as burrow ventilation [14].

## Supplementary Material

Data set: chironomidae impact on sediment respiration in the gradient of temperatures

## Data accessibility

Data are available as the electronic supplementary material.
